# Cerebral abscess following the self-extraction of teeth in patient with Ebstein’s anomaly: a case report

**DOI:** 10.1186/s12903-019-0893-3

**Published:** 2019-08-30

**Authors:** Soichiro Kawase, Yoshiyuki Okada, Kazushige Isono, Hitoshi Iwasaki, Takashi Kuno, Kohei Matsumura, Yiwen Fu, Yorikazu Harada, Tadashi Ogasawara

**Affiliations:** 1 Department of Special Care Dentistry, Matsumoto Dental University, Nagano, Japan; 2 Department of Special Care Dentistry, Graduate School of Biomedical and Health Sciences, Hiroshima University, 1-2-3 Kasumi, Minami-ku, Hiroshima, 734-8553 Japan; 3 University of California San Francisco, School of Dentistry, San Francisco, CA USA; 4 Division of Cardiovascular Surgery, Nagano Children’s Hospital, Nagano, Japan

**Keywords:** Brain abscess, Infective endocarditis, Antibiotic prophylaxis, Congenital malformation of the heart, Self-extraction of tooth, Intellectual disabilities

## Abstract

**Background:**

Antibiotic prophylaxis before invasive treatments, including dental extractions, is still recommended for patients at high risk of infective endocarditis. However, the risk from self-extraction of teeth in daily life of patients with intellectual disabilities is uncertain.

**Case presentation:**

A 6-year-old patient with Ebstein’s anomaly developed cerebral abscess, which appeared associated with infective endocarditis of dental origin. Two weeks after self-extraction of his deciduous teeth, he began to experience pain in his ear and developed continuous fever, followed by vomiting, facial spasm, and a loss of consciousness. He was admitted into a hospital for 2 months, during which he received intravenously administered antibiotics and a drainage tube in his brain.

**Conclusions:**

Deciduous teeth can be self-extracted before root resorption and natural shedding in patients with intellectual disabilities. When they are at high risk of infective endocarditis and frequently touch mobile deciduous teeth, it seems to be an option to extract the teeth early with antibiotic prophylaxis, rather than to wait natural fall.

## Background

Ebstein’s anomaly is a rare congenital malformation of the heart in which the tricuspid valve is displaced towards the ventricular apex. Severity of the disease varies from mild cases with no symptoms to serious cases with cardiac arrest and cyanosis with/without intellectual disabilities. When blood supply to the pulmonary artery is insufficient, both of the superior and inferior vena cavae are required to be reattached directly to the pulmonary artery with artificial blood vessels, forming the total cavo-pulmonary connection (TCPC) to avoid serious symptoms.

In 2007, the American Heart Association (AHA) reclassified patients who have undergone the Fontan surgery into a high risk group for infective endocarditis (IE) [1], and it recommended the use of antibiotic prophylaxis (AP) before any invasive procedures, including tooth extractions, gingival and apical procedures, and any other procedures involving perforation of the oral mucosa in them. Indeed, many retrospective studies have pinpointed the causative microbial agents of IE to be those of oral origin in 14–20% of the cases [2–5]. On the other hand, AP has been reported to prevent an exceedingly small number of cases of IE [6]. Thus, the AHA stated that AP was not recommended for dental events with minor bleeding in daily life, such as shedding of primary tooth and trauma to the lips and oral mucosa [1]. Moreover, the National Institute for Health and Clinical Excellence (NICE) stated that AP is no longer recommended for all dental procedures in their guidelines published in 2008 and updated in 2016 [7].

However, we encountered a young TCPC-treated patient with Ebstein’s anomaly who developed brain abscess which appeared associated with IE following self-extraction of his deciduous teeth before root resorption and natural shedding. We are reporting his case in order to provide important information regarding the appropriate timing of tooth extraction with AP during tooth transition period in high-risk children with intellectual disabilities.

## Case presentation

A six-year and 8-month old boy (height: 98 cm, weight: 13.4 kg) with Ebstein’s anomaly and intellectual disabilities; who had undergone the Glenn surgery at 10 days old to connect his superior vena cava to the pulmonary artery, and the Fontan surgery at 4 years old to connect his inferior vena cava to the pulmonary artery; began to experience sudden vomiting, a loss of consciousness, and facial spasm on August 8. He was taken by ambulance to a special medical institute at a children’s hospital, where he was immediately admitted into the intensive care unit.

The patient was previously diagnosed with rampant caries at the age of 5, and received conservative dental treatments for his 16 teeth with AP under general anesthesia because of high risk of IE, followed by regular checkups once every 3 months. At the regular dental checkup 3 months prior, his left lower deciduous central and lateral incisors were rigid without any mobility, although it was noted that the permanent successors existed under the gingiva behind these teeth. No dental problems were found except for fair to poor oral hygiene. The Plaque Index Silness and loe of the patient was 1.8 in average. It was reported that the patient began to frequently touch his left lower deciduous central and lateral incisors and he had self-extracted those teeth 2 weeks prior to the appearance of the reported symptoms. A week before his hospital admission, he complained of pain in his right ear and developed a continuous fever (> 38.0 °C). However, his parents did not consult his attending cardiologist because they had perceived these symptoms to resemble those of a common cold. In this time, they were not informed about the risk of IE associated with dental events or procedures.

**Fig. 1:**
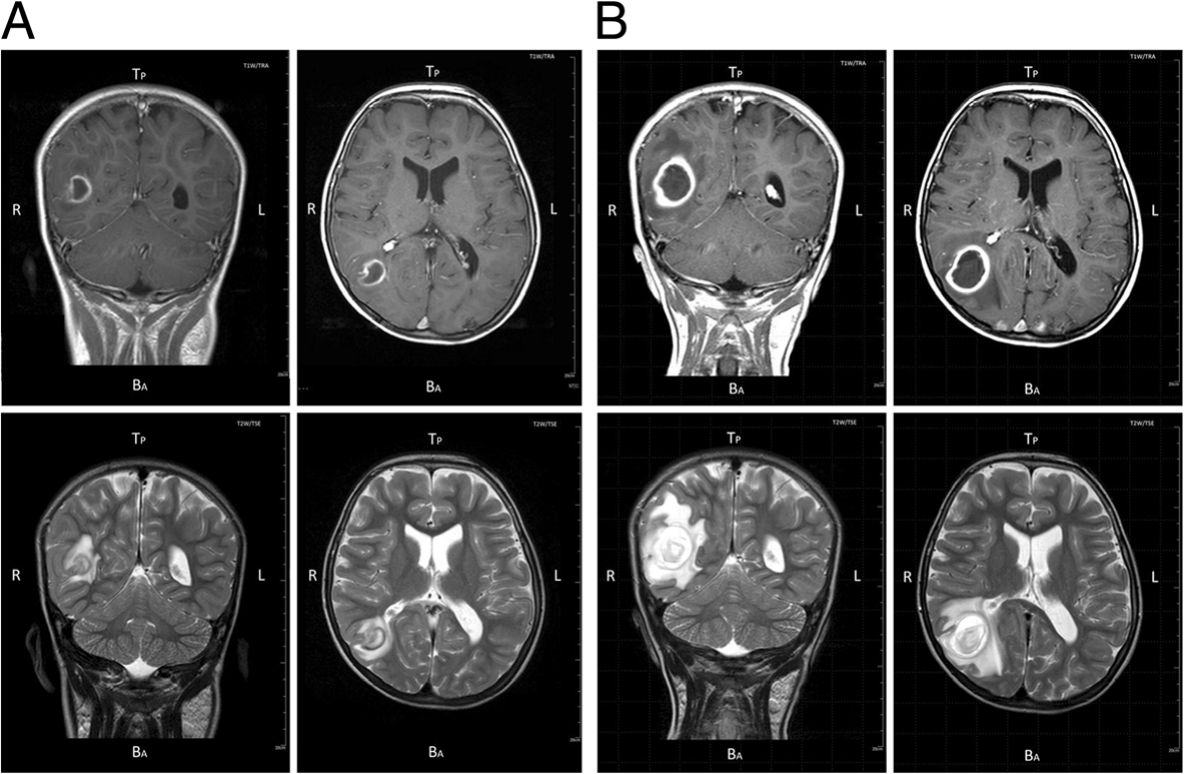
A magnetic resonance imaging of the brain demonstrating a ring-shaped abscess of 18 mm on August 8 (**a**) and that of 29 mm on August 22 (**b**)

**Fig. 2:**
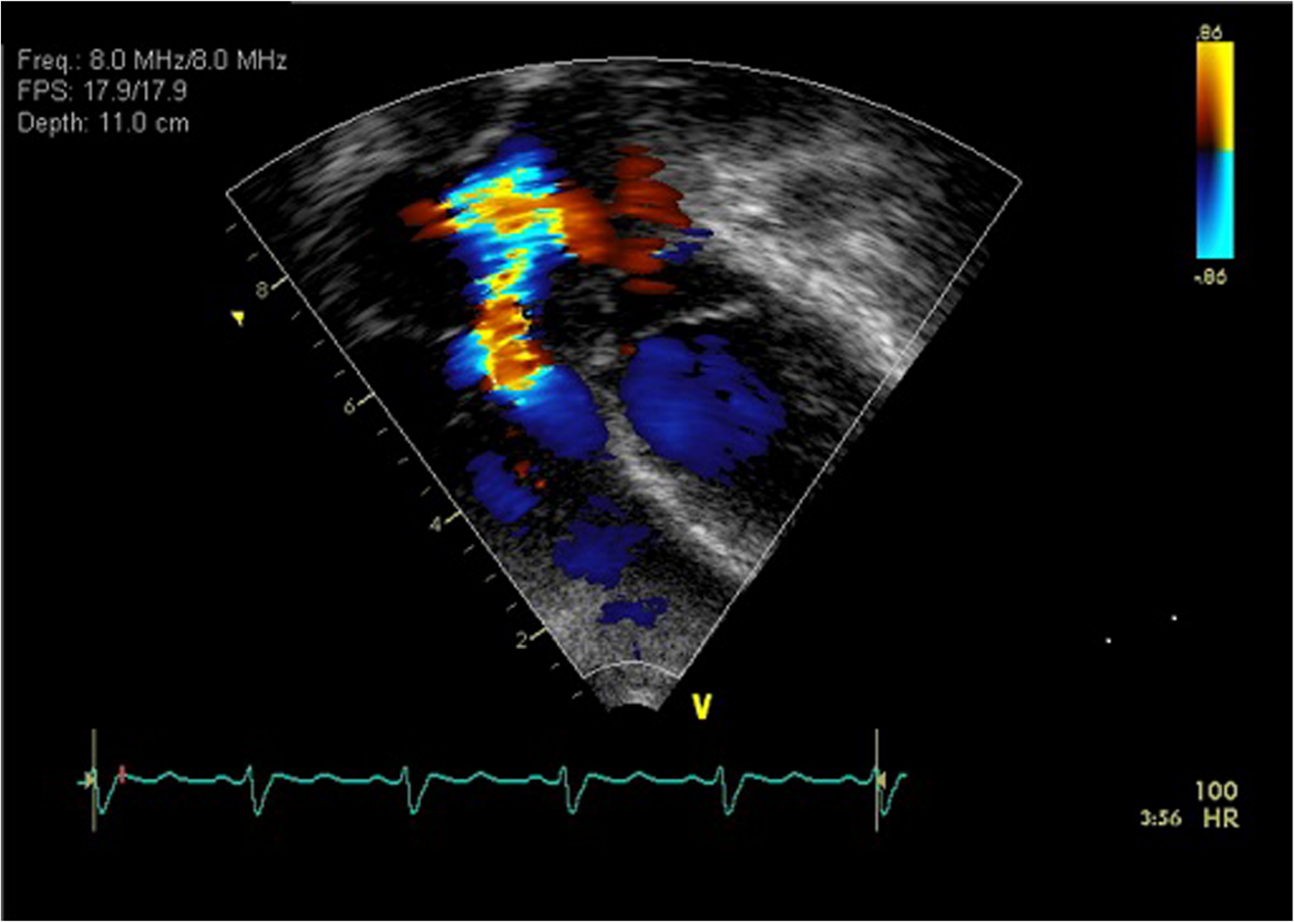
An echocardiographic image demonstrating an incomplete tricuspid atresia with moderate regurgitation in the patient

Both magnetic resonance imaging and computed tomographic (CT) scans of the brain were performed on admission and showed a ring-shaped abscess of 18 mm in diameter in the transitional area of the right temporal lobe (Fig. 1a). Echocardiography showed an incomplete tricuspid atresia with moderate regurgitation--predisposing heart conditions and minor echo criteria for the diagnosis of IE, but no oscillating intracardiac mass on valve or supporting structures, in the path of regurgitant jets, verrucous endocardial lesion, or thrombus in the heart--positive echo criteria of IE (Fig. 2). An electrocardiogram showed complete right bundle branch block and right axis deviation. A chest X-ray showed right ventricular hypertrophy (CTR = 58%).

As an initial therapy, the patient received 1) cefotaxime (200 mg/kg/day) and meropenem (120 mg/kg/day) intravenously from August 8 to16. However, in a brain scan on August 13, the abscess was found to have enlarged to 21 mm in diameter. In response, the attending doctor administered 2) rocephin (200 mg/kg/day) and meropenem (120 mg/kg/day) from August 17 to 21. On August 22, a third brain scan measured the size of the abscess to be 29 mm and showed that wall thickness of the abscess and the surrounding edema became more prominent (Fig. 1b) with continuous spasm. Subsequently, since the size of the abscess was still increasing and exceeding 2.5 cm while on antibiotics for 2 weeks, the doctor administered 3) panipenem/betamipron (95 mg/kg/day) from August 22 to 23, and a brain surgeon performed surgical drainage - craniotomy followed by approaching the brain mass and draining the pus out of the abscess by using CT-guidance to enhance the efficacy of antibiotic therapy and reduce raised intracranial pressure [8] on August 23, when the drugs effect reached its maximum. To find out the causes of insufficient effects from previously administrated antibiotics on a growing abscess, we obtained materials from the abscess. A Gram stain of them revealed gram-positive coccus, and blood and abscess culture tests detected intraoral bacteria of the *Streptococcus milleri* group. Based on the detection of these bacteria and possible involvement of the Methicillin-resistant *Staphylococcus aureus*, the doctor then administered 4) vancomycin (45 mg/kg/day) and rocephin (100 mg/kg/day) from August 23 to 29. Thereafter, the patient recovered steadily and began carbamazepine, an antiepileptic medication, to avoid post-operative epilepsy. Finally, he was discharged from the hospital after 2 months of treatment.

At a regular checkup after the reported incident, we informed the patient’s mother about the necessity of early extracting deciduous teeth with AP in her child, as oppose to waiting for their natural loss, to avoid self-extraction of his teeth before root resorption without AP. Afterward, the upper left and right deciduous central incisors and lateral incisors were extracted on January 31 and March 7 in the next year, respectively, under the use of AP with amoxicillin. We repeatedly disinfected sockets and checked for the appropriate healing process for 2 weeks after extractions. Although the rest of the deciduous teeth were extracted in the same way before their natural loss, no aberrant symptoms were observed during the course of that treatment.

## Discussion

Heart disease with incomplete tricuspid atresia with moderate tricuspid regurgitation observed by echocardiography (Fig. 2), the continuous fever (> 38.0 °C), and the presence of intraoral *Streptococcus milleri* group in the blood culture in the present case indicated possibility of IE according to the modified Duke criteria. The patient displayed the symptoms of IE, as well as brain abscess, within 2 and 3 weeks after the self-extraction of his teeth. Since the symptoms of IE are reported to develop within 2 weeks post bacteremia, followed by the attachment and proliferation of bacteria at the verrucous endocardial lesions [9], the process of this case suggested that the patient’s symptoms may have originated from bacteremia following the self-extraction of his teeth. The risk of a brain abscess caused by hematogenous spread from the infected heart must be much higher in patients with a high risk than those with a low risk of IE, while the risk of a brain abscess caused by direct expansion from dental infection should be the same between them. The brain abscess in the present case seemed to develop as a result of hematogenous spread from the heart infected via the socket of extracted teeth. In fact, some brain abscesses were reported to be caused by dental-related IE [10]. Furthermore, the *Streptococcus milleri* group was found in both the patient’s blood and the culture obtained from drainage of the brain abscess. Therefore, detection of common bacteria in oral flora, blood, and brain abscess of the patient may support the speculation of underlying cause.

Dental procedures are responsible for a fraction of cases of IE and AP may prevent only a small number of cases, even if AP were effective [6, 11]. Based on these results, in the most recent AHA and NICE guidelines, AP was no longer recommended for dental procedures that involves minor bleeding, such as deciduous tooth loss. Not surprisingly, the prevalence of IE did not increase for 2 years after such changes in the guidelines [12]. However, approximately 20% of physicians in the U.K. continued to use AP for treatments accompanied by bleeding in patients who are at high risk of IE [11]. This could have possibly masked the true risks of not using AP for the high risk group just after the changes. In fact, their most recent reports revealed a significant increase in IE incidence with a reduction in AP prescribing since introduction of the NICE guidelines [13] and update of AHA recommendations [14]. Recently, Lopez et al. [15] reported a case of a patient who died of a stroke attributable to IE that occurred 10 days after a dental procedure without AP. The patient had been treated with AP during every dental procedure up until the change in NICE guidelines. As seen in this recent and the current case reports, there are risks of IE and neurological complications associated with dental-related events, despite the low prevalence. There has not been past IE and neurological complications reported in these patients due to oral bleeding in daily activities, such as brushing and mastication, even though this type of bleeding in an over year causes a much higher frequency and cumulative duration of exposure to bacteremia than dental events with mild bleeding (e.g., shedding of deciduous teeth) [16, 17]. With such knowledge, self-extraction seems to have a greater risk for IE than natural deciduous tooth loss. It currently remains unknown the exact effects of bleeding time, number of bacteria from the bleeding region, and duration of bacteremia on causing IE. But the self-extraction of a tooth during transition period in the patient who takes warfarin potassium can cause enough bleeding leading to IE and brain abscess. Such event can also be comparable to normal tooth extraction, gingival and apical procedures, for which AHA has recommended the use of AP.

During the tooth transition period, both of the resorption of roots of deciduous teeth and the epithelization for permanent teeth suppress bleeding. Therefore, bacteremia and local infection are less likely to occur during natural shedding, and AP is considered unnecessary. In contrast, self-extraction of deciduous teeth before their root resorption evoke excessive bleeding, resulting in a comparable rate of bacteremia to regular tooth extraction. Previous animal experiments have proven that AP does prevent IE [18, 19], suggesting that AP can avoid the attachment and proliferation of bacteria at the endocardia in regular and self-extraction of teeth. Thus, the 6-year 8-month old patient in this case was very likely to develop IE due to the difficulty and lack of antibiotic administration for the self-extraction, and the infection may have spread to the brain where it formed an abscess.

Self-extraction of teeth in children with intellectual disabilities occurs often as a result of the probing of their own teeth, which cannot be completely avoided even under parental control. Since poor oral hygiene has been found to yield positive blood cultures with a bacteria level similar to that after tooth extraction [16, 20], self-extraction in conjunction with poor oral hygiene may elevate the risks for infection dramatically. As for the current case, the patient should have been informed that they need to visit the dental clinic when the tooth first became mobile to avoid self-extraction before root resorption and they need to consult his attending cardiologist after the appearance of aberrant symptoms. Furthermore, the dentist should have thoroughly assessed the mobility of the deciduous teeth more frequently during tooth transition period and chosen to perform early extractions with AP to avoid risks of bacteremia and ultimately IE in high risk patients. This can reduce the chances of self-extraction and developing IE and brain abscess.

## Conclusion

We encountered a 6-year and 8-month old patient with congenital heart disease who developed infectious endocarditis and a brain abscess after he self-extracted two deciduous teeth before root resorption. Children with congenital heart disease are at higher risks of infectious endocarditis which can be evoked through events as common as the self-extraction of deciduous teeth before its natural loss. Thus, to avoid such risks, when high-risk children with intellectual disabilities have mobile deciduous teeth, they should be encouraged to visit dental clinics for proper oral hygiene and early extraction, where antibiotic prophylaxis will be used to lower the probability of bacterial infection. Additionally, the current case emphasizes the importance of collaboration between cardiologists, their family members, and special care dentists in treating issues of tooth transition period in young patients with congenital heart disease.

## Data Availability

The datasets used and/or analysed for clinical practice and the current report are available from the corresponding author on reasonable request.

## Abbreviations

AHA: The American Heart Association
AP: Antibiotic prophylaxis
IE: Infective endocarditis
NICE: National Institute for Health and Clinical Excellence
TCPC: Total cavo-pulmonary connection
